# Time-course RNA-seq analysis reveals stage-specific and melatonin-triggered gene expression patterns during the hair follicle growth cycle in *Capra hircus*

**DOI:** 10.1186/s12864-022-08331-z

**Published:** 2022-02-16

**Authors:** Chun Li, Cong Feng, Guangyuan Ma, Shaoyin Fu, Ming Chen, Wenguang Zhang, Jinquan Li

**Affiliations:** 1 College of Animal Science and Technology, Inner Mongolia Minzu University, Tongliao, 028000 China; 2 College of Life Sciences, Zhejiang University, Hangzhou, 310058 China; 3 Inner Mongolia Academy of Agricultural & Animal Husbandry Sciences, Hohhot, 010018 China; 4 College of Life Science and Food Engineering, Inner Mongolia Minzu University, Tongliao, 028000 China; 5 College of Animal Science, Inner Mongolia Agricultural University, Hohhot, 010018 China

**Keywords:** Hair follicle growth cycle, Cashmere goat, RNA-seq, Melatonin, Signaling pathway

## Abstract

**Background:**

Cashmere goat is famous for its high-quality fibers. The growth of cashmere in secondary hair follicles exhibits a seasonal pattern arising from circannual changes in the natural photoperiod. Although several studies have compared and analyzed the differences in gene expression between different hair follicle growth stages, the selection of samples in these studies relies on research experience or morphological evidence. Distinguishing hair follicle growth cycle according to gene expression patterns may help to explore the regulation mechanisms related to cashmere growth and the effect of melatonin from a molecular level more accurately.

**Results:**

In this study, we applied RNA-sequencing to the hair follicles of three normal and three melatonin-treated Inner Mongolian cashmere goats sampled every month during a whole hair follicle growth cycle. A total of 3559 and 988 genes were subjected as seasonal changing genes (SCGs) in the control and treated groups, respectively. The SCGs in the normal group were divided into three clusters, and their specific expression patterns help to group the hair follicle growth cycle into anagen, catagen and telogen stages. Some canonical pathways such as Wnt, TGF-beta and Hippo signaling pathways were detected as promoting the hair follicle growth, while Cell adhesion molecules (CAMs), Cytokine-cytokine receptor interaction, Jak-STAT, Fc epsilon RI, NOD-like receptor, Rap1, PI3K-Akt, cAMP, NF-kappa B and many immune-related pathways were detected in the catagen and telogen stages. The PI3K-Akt signaling, ECM-receptor interaction and Focal adhesion were found in the transition stage between telogen to anagen, which may serve as candidate biomarkers for telogen-anagen regeneration. A total of 16 signaling pathways, 145 pathway mRNAs, and 93 lncRNAs were enrolled to construct the pathway-mRNA-lncRNA network, which indicated the function of lncRNAs through interacting with their co-expressed mRNAs. Pairwise comparisons between the control and melatonin-treated groups also indicated 941 monthly differentially expressed genes (monthly DEGs). These monthly DEGs were mainly distributed from April and September, which revealed a potential signal pathway map regulating the anagen stage triggered by melatonin. Enrichment analysis showed that Wnt, Hedgehog, ECM, Chemokines and NF-kappa B signaling pathways may be involved in the regulation of non-quiescence and secondary shedding under the influence of melatonin.

**Conclusions:**

Our study decoded the key regulators of the whole hair follicle growth cycle, laying the foundation for the control of hair follicle growth and improvement of cashmere yield.

**Supplementary Information:**

The online version contains supplementary material available at 10.1186/s12864-022-08331-z.

## Background

The Inner Mongolian cashmere goat (*Capra hircus*), an excellent cashmere goat breed in China, is famous for producing cashmere with superior quality and high yield. The cashmere goat has two different fibrous hair structures: thick and coarse hairs forming the guard layer and soft hairs forming the ground cashmere. The cashmere comes from secondary hair follicles (SHFs) in the skin [1], and the coarse hair comes from primary hair follicles [2, 3]. The cashmere obtained from goats is specially used for production of expansive textile products [4]. Under the periodic changes of the natural photoperiod, the growth of cashmere in the Inner Mongolian cashmere goat presents a seasonal pattern. Normally, a typical hair follicle growth cycle starts in July and stops in March of the following year with shedding of the cashmere at April [5].

Melatonin is an important mediator between photoperiod and the hair follicle growth. The cyclical fluctuation of melatonin levels directly affects the hair follicle growth [6]. Previous studies have shown that the use of exogenous melatonin could stimulate hair follicle growth. However, a number of studies have shown that the implantation and duration of melatonin may lead to earlier shedding of cashmere [6–9], or increase production by promoting the growth of cashmere [10, 11]. Although the positive role on cashmere growth of exogenous melatonin has been confirmed, previous experiments have not been able to dynamically show the gene expression profiles related to the whole cycle of hair follicle growth and the potential effect of exogenous melatonin on the hair follicle growth cycle.

The hair follicle growth cycle of the cashmere goat can be divided into three phases: anagen, catagen and telogen, which are regulated by specific genetic regulators [5]. For Inner Mongolian cashmere goats, the anagen phase of SHFs is from April to November, the catagen phase is from December to January, and the telogen phase is from February to March [12]. SHFs continue to grow during the anagen phase and the cashmere continues to elongate. At the catagen phase, hair follicle cells undergo apoptosis and the growth rate of cashmere slows down to stop. Finally, SHFs enter the telogen phase and are accompanied by the shedding of cashmere [13]. With the rapid development of high-throughput sequencing technology, some regulatory factors and signaling pathways involved in the hair follicle cycle have been found through differential expression and functional enrichment analysis. These well-known regulatory molecules and signaling pathways include Wnt/β- catenin [14–16], bone morphogenetic proteins (BMPs) [17, 18], sonic hedgehog (SHH) [19], notch [20], fibroblast growth factors (FGFs) [21], transforming growth factors (TGFs) [22] and keratin-associated proteins (KRTAPs) [5, 23], etc.

Long noncoding RNAs (lncRNAs) are a type of RNAs that are longer than 200 nucleotides but do not encode proteins. However, lncRNAs can regulate the expression of protein-coding genes at various levels to influence biological processes, including epigenetic regulation, transcriptional regulation and posttranscriptional regulation [24]. The regulation mechanisms of lncRNAs in hair growth have been reported by some recent studies. For example, several important hair follicle development signals (lncRNAs and mRNAs) are involved in primary wool follicle induction in carpet sheep [25]. Yin et al. [26] indicated that lncRNA-599,554 contributes the inductive property of dermal papilla cells in cashmere goat, which might be achieved through sponging chi-miR-15b-5p to promote the WNT3A expression. Wang et al. [27, 28] integrated analysis of lncRNA, miRNA and mRNA in cashmere goat skin during anagen and telogen stages and revealed potential ceRNA regulatory networks. Sulayman et al. [29] performed a comprehensive analysis of lncRNA and mRNA expression profiles during sheep fetal and postnatal hair follicle development and demonstrated that the interaction between lncRNA and their target genes may regulate the development of hair follicles. However, the roles of lncRNAs in controlling the whole hair follicle growth cycle have not been well described.

To clarify the regulatory mechanism of the hair follicle growth cycle and gain insight into the gene regulatory network perturbed by exogenous melatonin, we use RNA-seq analysis to investigate the expression patterns of seasonal changing genes (SCGs) among different gene clusters. The interactions between lncRNAs and mRNAs were also explored using co-expression network analysis. The monthly differentially expressed genes obtained from pairwise comparisons between the control and melatonin-treated groups were detected to identify the key regulators associated with the growth of secondary hair follicles and reveal potential signaling pathways which may be involved in melatonin-affected growth patterns.

## Results

### Transcriptome analysis and differential gene expression overview

**Fig. 1:**
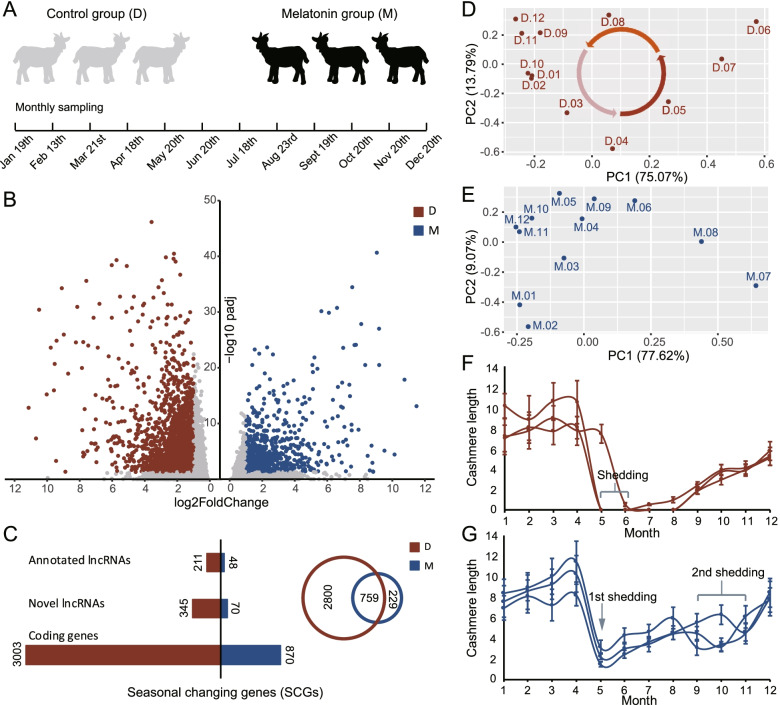
Overview of the transcriptome analysis. **A** The control and melatonin groups and sampling time. **B** Seasonal changing genes (SCGs) in the D and M groups. **C** The number of coding genes, novel lncRNAs and annotated lncRNAs in the D and M groups. The Venn plot indicates that 759 genes are detected in both D and M groups. **D** PCA plot of the median-normalized samples in the D group. The circle suggests the direction of samples in hair follicle growth stages. **E** PCA plot of the median-normalized samples in the M group. **F** The cashmere length change in the D group. The shedding occurs in May-June. **G** The cashmere length change in the M group. The first shedding occurs in May-June. Another shedding appears in September-November

A total of 72 samples spanning 12 months were analyzed in the control (D) and melatonin (M) groups (Fig. 1A). The experiments produced 2,959,842,480 clean reads in total (~926G). A total of 22,404 genes were detected from reads counting through the RNA-seq data analysis (Additional file 1). A total of 2365 novel lncRNA genes were identified after the protein-coding-potential test (Additional file 2). Using DESeq2 time-series data analysis, 3559 and 988 genes were subjected as SCGs by fold change ≥2 and adjusted p-value ≤0.05 in the D and M groups, respectively (Fig. 1B) (Additional file 3 and Additional file 4). Among these SCGs, a total of 345 novel lncRNAs and 211 annotated lncRNAs were detected in the D group. We found that SCGs in the M group were much less than those in the D group, and 76.8% (759) of the SCGs in the M group also existed in the D group (Fig. 1 C). According to the PCA diagrams in Fig. 1D and E, the samples of the D group showed a certain periodic distribution, but the sample distribution was disturbed after the melatonin treatment. It can be seen in Fig. 1F and G that the goats in two groups shed the cashmere in May, while goats in the M group had another shedding in September-November. An interesting finding is that in the M group, new cashmere has grown during the shedding in May, that is, the SHFs may not enter the telogen phase and the hair follicle growth cycle has restarted in advance (Fig. 1G).

### Stage-specific gene expression dynamics in the hair follicle growth cycle

**Fig. 2:**
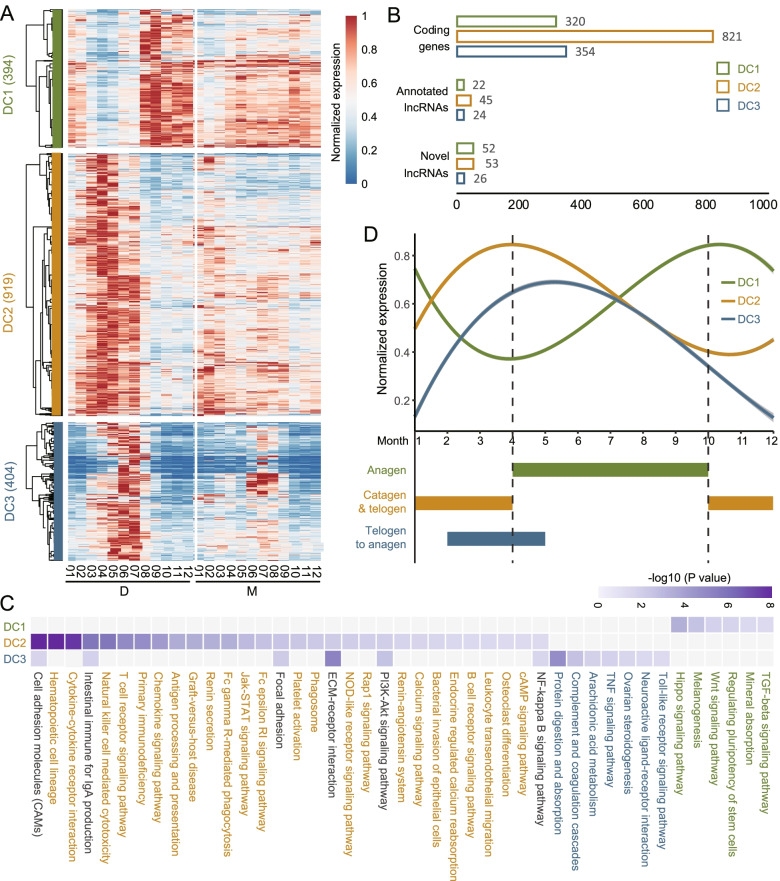
Stage-specific gene expression patterns and pathway enrichment. **A** Heatmap of the expression level of three D clusters (DC1, DC2 and DC3) in D and M groups. **B** The number of coding genes, annotated lncRNAs and novel lncRNAs in three D clusters. **C** KEGG pathway enrichment of the genes in DC1, DC2 and DC3. The pathways shared by DC2 and DC3 are shown in black. **D** The expression trends of three gene clusters. The hair follicle growth cycle is divided into three stages according to the expression patterns

The correct division of the hair follicle growth cycle is the basis for subsequent identification and analysis of regulatory factors. The existing studies are mostly based on morphological evidence or prior knowledge. A recent study divided the hair follicle growth cycle into anagen, catagen and telogen phases based on skin tissue sections and transcriptome data [5]. However, their analysis is based on static differential expression analysis, the dynamic change of gene expression has not been well studied. In our study, we analyzed the time-series transcriptome data covering the entire hair follicle growth cycle and obtained 3559 SCGs. Through WGCNA analysis, three key gene clusters (DC1, DC2 and DC3) that may be involved in the growth cycle of villi were identified in the D group (Fig. 2 A). The detailed genes of three clusters are listed in Additional file 5, which encompass 48.2% (1717) of the SCGs detected in the D group, including 91 annotated lncRNAs and 131 novel lncRNAs (Fig. 2B). Combining the expression patterns and functional enrichment results of three clusters (Fig. 2 A and C), we inferred the effects of these clustered genes on the hair follicle growth cycle. The genes in DC1 may promote cashmere growth and the genes in DC2 may be related to the regression of the SHFs. There may be an antagonistic relationship between DC1 and DC2, which can also be inferred from the gene expression trends in Fig. 2D. The gene expression in DC1 began to be up-regulated in April and reached a peak in October and started to be down-regulated, while the gene expression pattern in DC2 was exactly the opposite. In addition, it can also be found that the high expression of DC3 occurs when the expression of DC1 genes begins to be up-regulated and the expression of DC2 genes begins to down-regulate. Based on the above analysis, we divided the hair follicle growth cycle into three stages associated with three gene clusters: (1) anagen (April-October); (2) catagen and telogen (October-December and January-April); (3) telogen-anagen regeneration (February-May). The detailed functional analysis of these three clusters and their relationships with the hair follicle growth cycle are described below.

#### The DC1 cluster shows high expression in anagen progression stage

A total of 394 genes in DC1 (including 22 annotated lncRNAs and 52 novel lncRNAs) are positively correlated with the anagen progression stage (April-October), which may be involved in promoting the cashmere growth (Fig. 2 A and D). Some canonical pathways such as the Wnt, TGF-beta and Hippo signaling pathways, which have been proven to be closely related to hair growth [16, 30, 31] are also enriched in DC1 (Fig. 2 C) (Additional file 6). Three genes of the Wnt family WNT2/WNT2B/WNT11, together with their receptors FZD3 and FZD10 are involved in the regulation of the SHF development in cashmere goats. Lymphoid enhancer binding factor-1 (LEF1) is an essential transcription factor in the Wnt signaling, and it was strongly expressed during the anagen progression stage in this study. Its function in hair cell differentiation and follicle morphogenesis has already been discussed [32]. Genes in the TGF-beta signaling (BMP2, BMP8A, BAMBI and SMAD6) also showed similar patterns with the Wnt family. It is found that the genes enriched in the Hippo signaling overlap with those in the Wnt and TGF-beta signaling, indicating the crosstalk between Wnt and TGF-beta signaling formed by the joint regulation of downstream pathways [16, 33]. In addition, some downstream regulatory mechanisms of hair growth and cycling were also confirmed in this study. For example, the homeobox transcription factor DLX3 and one of its regulating genes, HOXC13, were also highly expressed in the anagen stage. The regulatory cascade positions DLX3 downstream of Wnt signaling and regulates other transcription factors related to hair follicle (HF) differentiation (such as HOXC13) [34]. KRTs (KRT26, KRT35, KRT36, KRT39, KRT6A, KRT74 and KRT84) and KRTAPs (KRTAP3-1 and KRTAP11-1) in DC1 were also associated with HF development [35]. SHH and its receptor PTCH2 in the sonic hedgehog (Shh) signaling pathway were also found in DC1. The function of Shh signaling is indicated as an essential regulator for controlling ingrowth and morphogenesis of the HFs [36–38], but it is not necessary for initiating the HF development [36].

#### The DC2 cluster prefers to be highly expressed in catagen and telogen

A total of 919 genes in DC2 (including 45 annotated lncRNAs and 53 novel lncRNAs) were up-regulated from October to April of the following year (Fig. 2 A and 2D), which corresponded to the catagen and telogen phases of the hair follicle growth cycle. It should be pointed out that due to the limitation of sampling interval, the catagen and telogen stages were not further distinguished in this study. The expression pattern of DC2 is negatively correlated with DC1, suggesting that there may be an antagonistic relationship between the genes in DC2 and DC1. DC2 genes are mainly enriched in pathways such as Cell adhesion molecules (CAMs), Cytokine-cytokine receptor interaction, Jak-STAT, Fc epsilon RI, NOD-like receptor, Rap1, PI3K-Akt, cAMP, NF-kappa B and many immune-related pathways (Fig. 2 C) (Additional file 6). A previous study also found that differentially expressed genes between anagen and telogen SHF-derived dermal papilla cells of the cashmere goats were also enriched in CAMs and Cytokine-cytokine receptor interaction pathways [1]. A number of interleukin (IL) superfamily genes were involved in the enriched pathways of DC2, such as the IL1 family (IL7 and IL18), IL10, IL15, and IL receptors (IL2RG, IL3RA, IL6R, IL7R, IL11RA and IL20RA). The CC chemokine subfamily (CCL5, CCL21, CCL22 and CCL26) and receptors (CCR4 and CCR6) were also found in the DC2 cluster. These chemokines are mainly involved in cell migration, immunity and inflammation [39]. During the HF regression, these chemokines may guide the migration of immune cells such as dendritic cells [40] and Regulatory T cells [41], thereby regulating the immune response to apoptotic cells [42]. The JAK3 and STAT4 genes in the JAK-STAT signaling were highly expressed in catagen and telogen, which have been found to maintain HF stem cell quiescence and inhibit hair growth [43–45]. In addition, Dickkopf1 (DKK1), a Wnt signaling inhibitor, was also found in DC2. DKK1 has been strongly suggested to promote regression of HFs by suppressing Wnt/β-catenin signaling and inducing apoptosis in follicular keratinocytes [46, 47].

#### The DC3 cluster is specifically expressed in telogen-anagen regeneration stage

DC3 cluster contains a total of 404 genes (including 24 annotated lncRNAs and 26 novel lncRNAs) and shows specific high expression during the transition period from February to May (Fig. 2 A and D). Due to the overlap of the early anagen and telogen-anagen regeneration stages, the genes of DC3 and DC2 were partially enriched in several same pathways like CAMs, Focal adhesion, extracellular matrix (ECM)-receptor interaction, PI3K-Akt and NF-kappa B signaling (Fig. 2 C) (Additional file 6). The PI3K-Akt signaling has been proved to be essential for HF regeneration [48, 49]. A previous study found that the Toll-Like Receptor 3 (TLR3) activated by a dsRNA was able to promote HF regeneration [50]. Several collagen genes (COL1A1, COL1A2, COL3A1, COL5A2, COL6A3, COL6A5 and COL6A6) in the PI3K-Akt signaling, ECM-receptor interaction and Focal adhesion were found in DC3. These collagen genes may serve as candidate biomarkers for telogen-anagen regeneration. For example, a kind of self-assembling peptide hydrogel scaffold was used to build the ECM environment in vitro to promote HF regeneration [51].

### Pathway crosstalk through mRNA-lncRNA co-expression network in hair follicle growth cycle

**Fig. 3:**
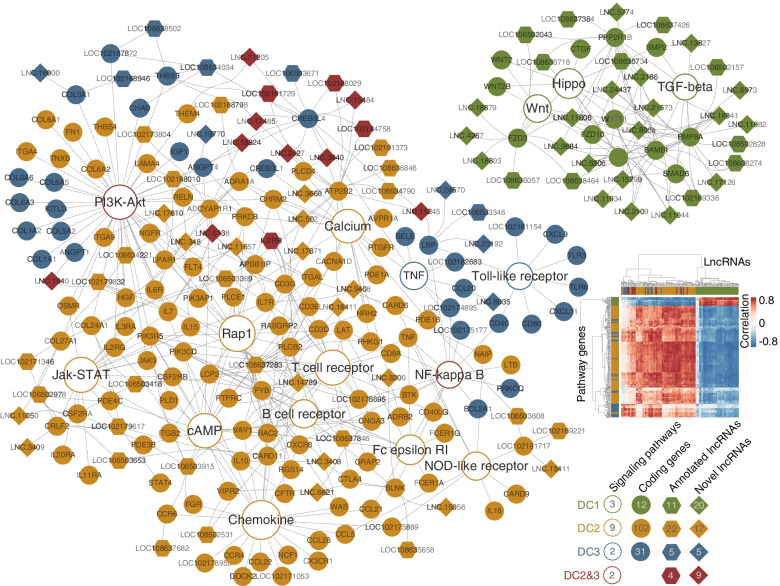
Pathway-mRNA-lncRNA co-expression network. A total of 16 signaling pathways, 143 pathway genes, and 93 co-expressed lncRNAs (Pearson correlation ≥0.8) are involved in this network. The size of the pathway node is positively related to the number of genes connected to this pathway

A total of 16 signaling pathways, 145 pathway genes, and 93 co-expressed lncRNAs (Pearson correlation ≥0.8) (Additional file 7) are enrolled in this pathway-mRNA-lncRNA network (Fig. 3). It can be seen that the DC1 subnet has no positive correlation with DC2 and DC3, which can be explained by the distinct expression patterns in Fig. 2D and indicates possible antagonistic relationship between DC1 and DC2. The telogen-anagen transition phase of DC3 overlaps with the telogen stage, so the DC3 subnet is closely connected to DC2. Through this network, the function of lncRNAs can be inferred by their co-expressed coding genes. Three novel lncRNAs (LNC.6206, LNC.8064 and LNC.16,941) were highly connected (by more than 6 coding genes) in the DC1 subnet, indicating that they may mediate in promoting HF development. Similarly, four novel lncRNAs (LNC.348, LNC.6138, LNC.11,657 and LNC.14,789) and two annotated lncRNAs (LOC106503915 and LOC108637283) were found with high degrees in the DC2 subnet. The coding genes connected to them cover all the signaling pathways in DC2, indicating that these lncRNAs are likely to be involved in the SHF degeneration. The PI3K-Akt and NF-kappa B signaling are both functioning in DC2 and DC3 subnets. We found nine novel lncRNAs and four annotated lncRNAs connecting DC2 and DC3, which may play a role in the transition from telogen to early anagen.

### Expression differences triggered by melatonin reveal a potential signal pathway map regulating cashmere growth

**Fig. 4:**
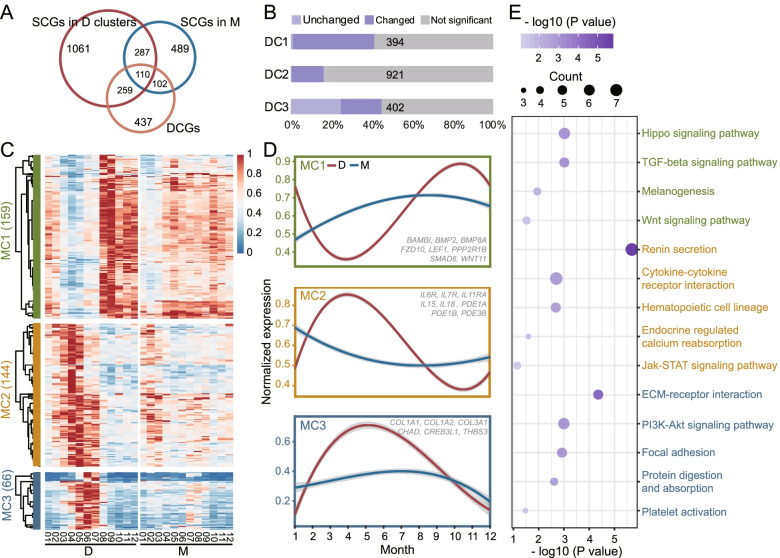
Analysis of differences in the expression patterns triggered by melatonin. **A** Venn diagrams showing SCGs in D clusters and M group and DCGs between D and M groups. **B** The changed and unchanged genes of three D clusters after melatonin treatment. **C** Heatmap of the expression level of three M clusters (MC1, MC2 and MC3) in D and M groups. **D** The expression patterns of three M clusters in D and M groups. **E** KEGG pathway enrichment of the genes in MC1, MC2 and MC3

A total of 908 genes (including 70 novel lncRNAs and 48 annotated lncRNAs) were detected to have different expression patterns between the D group and the M group (Fig. 4 A) (Additional file 8). When treated with melatonin, 80 (24.6%) genes in DC3 maintained the same pattern as group D, but the expression patterns of most genes in DC1 and DC2 have changed (Fig. 4B). Among the 908 differential changing genes (DCGs), 369 genes belonging to the three clusters DC1, DC2 and DC3 were divided into three clusters MC1 (159), MC2 (144) and MC3 (66), respectively (Fig. 4C) (Additional file 9). After melatonin treatment, MC1 and MC2 in the M group lost the periodic expression fluctuations as in the D group (Fig. 4D). In April-May, the gene expression of MC1 dropped to the lowest while the expression of MC2 rose to the highest in the D group and shedding appeared. Pathway enrichment showed that MC1 and MC2 were involved in the promotion and regression of HFs, respectively (Fig. 4E) (Additional file 10). The six novel lncRNAs in the DC1 subnet (LNC.20,921, LNC.13,171, LNC.15,017, LNC.18,879, LNC.18,803 and LNC.20,565) disappeared in MC1, while LNC.8064 and LNC.16,941 may still play a role in the cashmere growth under melatonin treatment. In the M group, the expression of MC1 was still rising and MC2 was falling from January to April, which may lead to a non-resting period after shedding in the M group. Therefore, genes in MC3 (including LNC.15,245), which may be responsible for restarting HF growth, did not show a significant increase in expression in February-May. In September-November, the expression of MC1 in the M group was relatively lower than that in the D group, while the expression of MC2 in the M group was higher than that in the D group, which may disrupt the growth and maintenance of HFs and cause a local shedding.

**Fig. 5:**
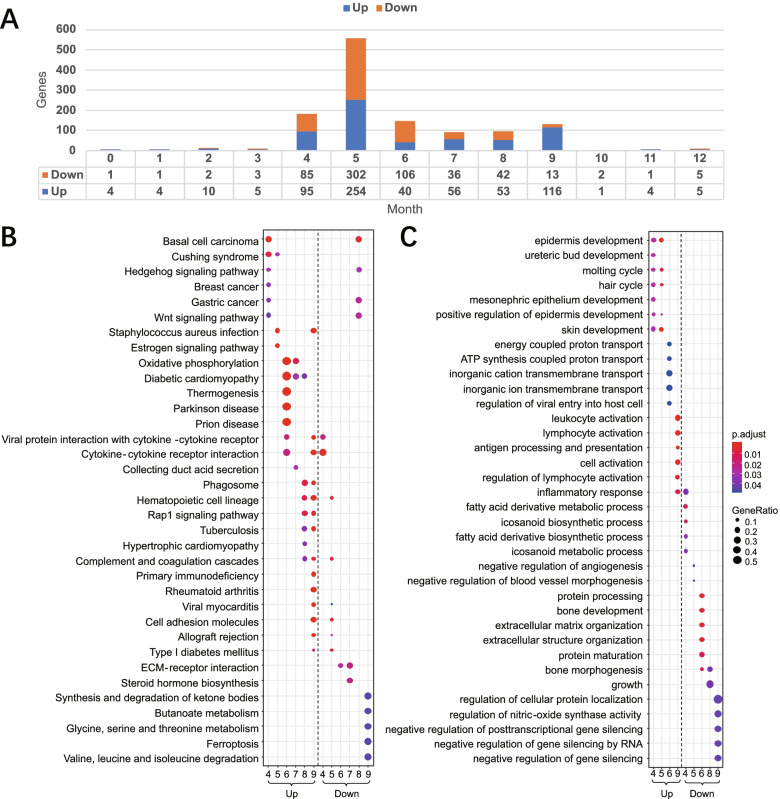
Detection of monthly differential expressed genes (DEGs) triggered by melatonin and pathway enrichment analysis. **A** The number and distribution of monthly differential expressed genes triggered by melatonin between M and D group. **B** The KEGG pathway enrichment analysis of monthly differential expressed genes. **C** The GO biological process enrichment analysis of monthly differential expressed genes. Note that up-regulated and down-regulated genes in different months are separated for GO & KEGG analysis, in which ‘Up’ represents up-regulated genes, and ‘Down’ represents down-regulated genes. The color of nodes represents adjusted p-value, and the size of nodes represents the ratio of gene numbers

To reveal the differential expression pattern every month, we used DESeq2 to obtain monthly differentially expressed genes (monthly DEGs) between melatonin and control groups with adjusted p-value (padj) ≤0.05 and |log2FoldChange| ≥1. A total of 941 monthly DEGs were identified from monthly pairwise comparisons. The monthly DEGs are mainly distributed from April and September (Fig. 5 A), which exactly cover the whole anagen stage, and 906 (96%) of them are protein-coding genes. The KEGG analysis results (Fig. 5B) (Additional file 11) showed that Hedgehog related genes (SHH, PTCH2, PTCH1) and Wnt related genes (FZD10, WIF1, LEF1, WNT11) were up-regulated in April, while the GO results (Fig. 5 C) (Additional file 12) showed that Hedgehog genes (PTCH2, FOXE1) and other related up-regulated genes like FOXN1, HOXC13, KRT25 and KRT71 were significantly enriched from April to May. Related studies have shown that Wnt [52–54] and Shh [36, 55] signaling can promote hair follicle cell division and the initiation of anagen. Potter et al. [56] demonstrated that nude mutant gene FOXN1 is a regulatory target of HOXC13 and the knockdown of both genes could cause abnormal hair growth. Yu et al. [57] showed that a missense mutation at the helix terminus of KRT25 can cause a reduction of woolly hair. KRT71 is an inner root sheath keratin, and the mutant of KRT71 can disrupt keratin intermediate filament formation [58]. Therefore, the Hedgehog related genes (SHH, PTCH2, PTCH1, FOXE1), Wnt related genes (FZD10, WIF1, LEF1, WNT11), and other hair development related genes FOXN1, HOXC13, KRT25 and KRT71 may be responsible for the initiation of a fast anagen progressing stage from April to July. Meanwhile, the KEGG results (Fig. 5B) showed that the expression of ECM receptor interaction genes (COL6A3, THBS3, COL1A1, FRAS1, FREM1/2) was downregulated from June to July. ECM is an important matrix required for hair follicle cell growth. Xu et al. [59] showed that the amount of ECM occupied by each cell determined the volume size of dermal papilla (DP) in hair follicles, and Zhu et al. [1] demonstrated that the rapid growth of hair follicles at the anagen phase in cashmere goat required high expression of ECM and cell surface proteins. The down-regulation of ECM-related genes from June to July may contribute to the inhibition of hair follicle growth, and the down-regulation of genes involved in the Hedgehog (SHH, PTCH2) and Wnt (WNT6, NOTUM, SFRP2) pathways indicated that hair follicle growth may be inhibited in August. The expression of genes in chemokine signaling pathway (CCL17, CCL22, CCL2, LYN, RAC2, LOC102170772, PIK3CG, VAV1) was up-regulated in September. Experiments by Nagao et al. [40] have shown that chemokines can induce immune cell migration as the hair follicle enters the catagen phase, which may further promote the hair follicle apoptosis. Therefore, the downregulated ECM receptor interaction genes (COL6A3, THBS3, COL1A1, FRAS1, FREM1, FREM2), Hedgehog genes (SHH, PTCH2), Wnt genes (WNT6, NOTUM, SFRP2) and up-regulated chemokines (CCL17, CCL22, CCL2, LYN, RAC2, LOC102170772, PIK3CG, VAV1) may contribute to the second cashmere shedding happened from August to November. A transcriptome analysis of the melatonin-treated group with MTC knockdown experiments confirmed that melatonin can promote hair follicle development by activating the NF-kappa B pathway through promoting the expression of MTC [60]. Genes in NF-kappa B signaling pathway (CD40LG, LTB, LOC102176695, LYN, BTK) were up-regulated in September, which may contribute to the hair follicle development after the second cashmere shedding. The representative GO & KEGG enrichment results of monthly DEGs mentioned above were summarized in Table 1.

**Table 1:** Summary of representative GO & KEGG enrichment of monthly DEGs

Cluster	Description	p.adjust	Gene Name
Month4_Up	hair cycle	0.0276	PTCH2, FOXE1, HOXC13, FOXN1
Month5_Up	hair cycle	0.0082	PTCH2, FOXE1, FOXN1, HOXC13, KRT71, KRT25
Month4_Up	Hedgehog signaling pathway	0.0306	SHH, PTCH2, PTCH1
Month4_Up	Wnt signaling pathway	0.0428	FZD10, WIF1, LEF1, WNT11
Month6_Down	ECM-receptor interaction	0.0259	COL6A3, THBS3, FREM1, COL1A1
Month7_Down	ECM-receptor interaction	0.0218	FREM1, FRAS1, FREM2
Month8_Down	Wnt signaling pathway	0.0257	WNT6, NOTUM, SFRP2
Month8_Down	Hedgehog signaling pathway	0.0318	SHH, PTCH2
Month9_Up	Chemokine signaling pathway	0.0002	CCL17, CCL22, CCL2, LYN, RAC2, LOC102170772, PIK3CG, VAV1
Month9_Up	NF-κB signaling pathway	0.0020	CD40LG, LTB, ZAP70, LYN, BTK

Note: The first two lines are derived from GO BP results and others are derived from KEGG results. P.adjust represents the p-values adjusted using the Benjamini & Hochberg method

## Discussion

In this study, the cashmere goat skin samples of the experimental group and the control group covered 12 months, that is, the entire hair follicle growth cycle. Many studies have used RNA-seq to explore the differences in gene expression in different growth stages of cashmere. For example, Geng et al. [61] conducted a functional analysis of the differences in gene expression between three developmental stages of hair follicles in cashmere goats, and identified key genes that are involved in the regulation of cashmere growth. Zhang et al. [13] performed transcriptome sequencing analysis on hair follicles in four seasons and explored the regulation of seasonal variation genes on the hair follicle growth cycle of the cashmere goat and milk goat. However, these studies only selected three or more stages determined by experiments or experience at the cellular level.

The gene expression pattern for 12 months can provide useful information for distinguishing different cashmere growth stages from the genetic and molecular levels. According to the cluster-month correlations in Fig. 2 C, we grouped the hair follicle growth cycle into three main stages: (1) anagen (April-October); (2) catagen and telogen (October-December and January-April); (3) telogen-anagen regeneration (February-May). The corresponding gene clusters are DC1, DC2 and DC3, respectively. Some canonical pathways such as the Wnt, TGF-beta and Hippo signaling pathways are enriched in DC1. DC2 genes are mainly enriched in pathways such as Cell adhesion molecules (CAMs), Cytokine-cytokine receptor interaction, Jak-STAT, Fc epsilon RI, NOD-like receptor, Rap1, PI3K-Akt, cAMP, NF-kappa B and many immune-related pathways. Interestingly, due to the overlap of the early anagen and telogen-anagen regeneration stages, the genes of DC3 and DC2 were partially enriched in several same pathways like CAMs, Focal adhesion, extracellular matrix (ECM)-receptor interaction, PI3K-Akt and NF-kappa B signaling. Besides, by constructing a co-expression network of genes (that are enriched in key pathways) and lncRNAs in three clusters, we reveal the possible regulators for crosstalk between different signaling pathways, and unearthed novel lncRNAs that may participate in these pathways. For example, in the DC1 subnet, the Wnt and Hippo pathways are connected by six protein-coding genes (WNT2, WNT2B, WNT11, FZD3, FZD10, and LEF1), while Wnt and TGF-beta are cross-linked by the key BAMBI gene. The crosstalk between these three pathways has been proven to play an important role in cell proliferation and differentiation [33]. In addition, long non-coding RNAs (four known and nine novel) were found to bridge between DC2 and DC3 subnets, which may play a role in the transition from telogen to early anagen. One of the nine novel lncRNAs, LNC.15,245, forms a crosstalk between six pathways (PI3K-Akt, TNF, Calcium, cAMP, NF-kappa B and Toll-like receptor) by connecting three protein-coding genes (CREB3L4, ATP2B2 and LBP).

In addition to exploring the gene expression regulation mechanisms of the transition between different stages of the hair follicle cycle, this study also helps to unravel the role of exogenous melatonin in the specific stages of the hair follicle growth cycle. By identifying genes that exhibit different expression patterns during the hair follicle growth cycle under the stimulation of melatonin, we also obtained three gene clusters (MC1, MC2 and MC3) that may affect the hair follicle growth cycle. Among them, MC1 genes (BAMBI, BMP2, BMP8A, FZD10, LEF1, PPP2R1B, SMAD6 and WNT11) and MC2 genes (IL6R, IL7R, IL11RA, IL15, IL18, PDE1A, PDE1B and PDE3B) showed opposite periodicity in group D. However, after the melatonin treatment, this regular fluctuation has been disordered. MC3 genes (COL1A1, COL1A2, COL3A1, CHAD, CREB3L1 and THBS3) were expressed specifically in the anagen restart phase (Apr-May) in group D, but there was no similarly significant expression pattern in group M.

The relative expression levels of monthly DEGs (Additional file 13) showed that the hair development related genes HOXC13, KRT25, KRT71, FOXN1 were generally expressed at higher levels at the beginning of fast anagen progressing period from April to May, implying that they may function to promote the initiation of anagen. Wnt genes (Wif-1, WNT11, FZD10, LEF1, NOTUM, SFRP2, WNT6) together with Hedgehog genes (SHH, PTCH1, PTCH2, FOXE1) showed higher expression levels between April and May, but decreased in August, which implied that Wnt-related genes may promote the rapid transition into anagen phase of hair follicles between April and May, and repress the growth of hair follicles on the eve of the second cashmere shedding period in August. Chemokines (CCL17, CCL22, CCL2, LYN, RAC2, LOC102170772, PIK3CG and VAV1) and NF-κB genes (ZAP70, LYN, BTK, CD40LG, LTB) were highly expressed in September. The NF-κB pathway may facilitate the progress of the subsequent cashmere growth phase. Meanwhile, chemokines such as LTN may promote the second cashmere shedding.

**Fig. 6:**
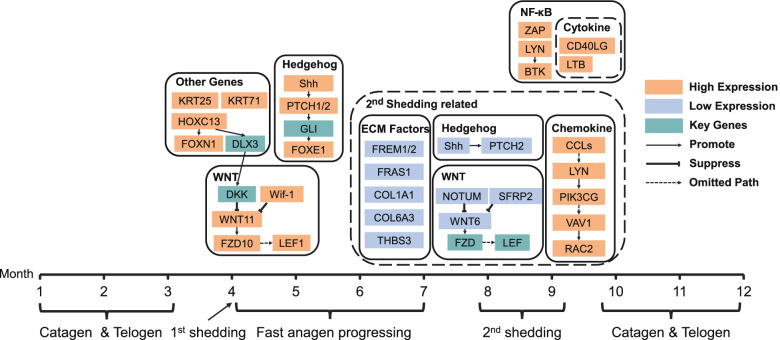
The putative pathway regulation model of the hair follicle growth cycle triggered by melatonin. The signal pathways related to cashmere shedding are marked with dotted line boxes, and the other solid line boxes mark the signal pathways which may promote the hair follicle development

KEGG pathway could be used as a reference to demonstrate the regulatory relationships of differentially expressed genes. Taking the above results together and collating the relevant KEGG pathway visualization results (Additional file 14), here we proposed a signaling pathway diagram of melatonin influenced hair follicle growth cycle (Fig. 6), which covered the main differentially expressed genes related to cashmere growth in anagen phase from April to September. The anagen phase of melatonin-treated groups was composed of a fast anagen progressing stage and a second cashmere shedding stage. The fast anagen progressing stage was from April to July, and this phase was characterized by the occurrence of the first massive cashmere shedding at the end of April, and the rapid transition into anagen phase of hair follicles from May to July, when the quick resumption of cashmere growth appeared instead of residing in a resting period. The rapid resumption of the anagen phase of hair follicles may be due to the high expression of KRT25, HOXC13 and HOXC13’s regulatory target FOXN1, high expression of FZD10, WIF1, LEF1, WNT11 in Wnt signaling pathway, and SHH, PTCH1, PTCH2, FOXE1 in sonic hedgehog signaling pathway. The second cashmere shedding period was from August to September. The appearance of the second cashmere shedding may not only be associated with the low expression of ECM signaling molecules such as FREM1, FREM2, FRAS1, COL1A1, COL6A3, THBS3 in June and July, sonic hedgehog signaling pathway genes such as SHH, PTCH2 and WNT signaling pathway genes such as NOTUM, SFRP2, WNT6 in August, but also with the high expression of chemokines such as CCL, LYN, PIK3CG, VAV1, RAC2 in August. In addition, the highly expressed genes in NF-kappa B signaling pathway such as CD40LG, LTB, ZAP, LYN, BTK in September may promote the subsequent growth of cashmere after the second cashmere shedding period.

## Conclusions

In summary, this study systematically analyzed RNA-seq data from skin samples of cashmere goats covering the entire hair follicle growth cycle and identified a series of key regulators (including genes and lncRNAs) that may be involved in the cashmere growth processes. Based on gene expression patterns, we elucidated a more precise division of the hair follicle growth cycle from the molecular level. Some canonical signaling pathways such as Wnt, TGF-β, Jak-STAT, NF-κB are detected in each phase of the hair follicle growth cycle. Pairwise comparisons between the control and treated groups every month reveal a potential signaling pathway map (including Wnt, Hedgehog, ECM, Chemokines and NF-κB signaling pathways) affecting the growing stage triggered by melatonin. Our study decodes the key regulators of the whole growth cycle of the hair follicles, laying the foundation for the control of cashmere growth and improvement of cashmere yield.

## Methods

### Skin sample and cashmere collection

Six female, half-sib, Inner Mongolia cashmere goats born on March 2013 with an initial weight of 33.35±1.93 kg were randomly divided into two groups: control group (D) and melatonin group (M). Three cashmere goats in the experimental group were implanted with melatonin (2 mg/kg BW) every two months since December 2014. Melatonin was subcutaneously implanted at the base of the ear. Skin and cashmere samples from the six cashmere goats were collected monthly (Jan 19th, Feb 13th, Mar 21st, Apr 18th, May 20th, Jun 20th, Jul 18th, Aug 23rd, Sept 19th, Oct 20th, Nov 20th, and Dec 20th ) in a 10-cm^2^ patch on the right mid-side of the body (behind the scapula). The subsequent skin and cashmere samples were taken as close as possible to the previous collection site, and the skin incisions were allowed to self-heal with application of the Yunnan Bai Yao (Chinese medicine: Z53020798). Immediately after collection, all skin samples were frozen in liquid nitrogen and stored at −80 °C until use. At each sampling time point, 50 cashmeres (hairs from SHFs) were taken from each goat to measure the cashmere length.

### RNA extraction, library preparation and sequencing

Total RNA from 72 collected skin samples were isolated using the TRIzol^TM^ reagent (Invitrogen, USA) following the manufacturer’s instructions (https://assets.thermofisher.com/TFS-Assets/LSG/manuals/trizol_reagent.pdf). RNA purity was verified using the NanoPhotometer® spectrophotometer (Implen, USA) (https://www.implen.de/wp-content/uploads/docs/Implen-NanoPhotometer-User-Manual-N120-NP80-N60-N50-C40.pdf). The ribosomal RNA (rRNA) was removed first by the Epicentre Ribo-zero^TM^ rRNA Removal Kit (Epicentre, USA) from 3 µg total RNA to prepare the RNA library. The sequencing libraries were generated using the NEBNext® Ultra^TM^ Directional RNA Library Prep Kit for Illumina (NEB, USA) following manufacturer’s instructions. Finally, the strand-specific libraries were sequenced on the Illumina Hiseq 4000 system (paired-end 150 bp reads).

### Quality control

FastQC (https://www.bioinformatics.babraham.ac.uk/projects/fastqc/) is used to filter out low-quality residues and joints of raw reads produced by RNA-seq. The quality of clean reads (Q20, Q30, and GC content) were detected. The high quality clean reads were retained for subsequent analysis.

### Read alignment and transcriptome assembly

Clean reads were aligned to the goat genome (NCBI assembly ARS1) using HISAT2 [62] under default settings. The mapped reads of each sample were assembled by StringTie [62] in a reference-based mode. All transcripts assembled were merged into a Gene transfer format (GTF) file.

### Novel lncRNA identification

The transcripts without matching the known annotations were selected to identify novel lncRNAs through the following steps: (1) transcripts less than 200 bp in length were removed; (2) transcripts with exon number less than 2 were removed; (3) the coding potential of each transcript was predicted using CPC2 [63] and CNCI [64], and transcripts predicted as “noncoding” were imported into Pfam Scan (http://ftp.ebi.ac.uk/pub/databases/Pfam/Tools/) to filter out those with known protein family domains (Pfam release 30).

### Reads counting and time-series differential expression analysis

All coding genes and lncRNA genes were included to calculate the count matrix using htseq-count [65]. The likelihood ratio test (LRT) in DESeq2 [66] was used to identify the seasonal changing genes (SCGs) in both control and melatonin group with model “~ time” versus “~ 1”. The SCGs were defined as genes with significant expression changes throughout the hair follicle growth cycle. Initially, the count matrix was normalized by the size factors estimated by DESeq2 and the median of the normalized gene expression values at each time point were used to calculate the fold change${f}_{i}$:

$${\varvec{\mu }}_{\varvec{i}\varvec{t}}={\varvec{m}\varvec{e}\varvec{d}\varvec{i}\varvec{a}\varvec{n}}_{\varvec{j} }\left({\varvec{G}}_{\varvec{i}\varvec{j}\varvec{t}}\right)$$

$${\varvec{f}}_{\varvec{i}}= \frac{{\varvec{m}\varvec{a}\varvec{x}}_{\varvec{t} }\left({\varvec{\mu }}_{\varvec{i}\varvec{t}}\right)}{{\varvec{m}\varvec{i}\varvec{n}}_{\varvec{t} }\left({\varvec{\mu }}_{\varvec{i}\varvec{t}}\right)+1}$$

Where${G}_{ijt}$is the normalized expression value of gene$i$in goat$j$at sampling time$t$. The thresholds for adjusted p-value and fold change were set to 0.05 and 2, respectively. Besides, the differential changing genes (DCGs) between control and melatonin group were detected using LRT with model “~ time + condition + time: condition” versus “~ time + condition”.

### Gene cluster detection

The SCGs detected in control group were included for weighted gene co-expression network analysis (WGCNA) [67]. Pearson correlations between gene clusters and trait data (months) were calculated. The minimum cluster size was set to 50, and clusters with a tree height less than 0.25 in the dendrogram were merged.

### Pathway enrichment

The DAVID database [68] was used to identify the significantly enriched Kyoto Encyclopedia of Genes and Genomes (KEGG) pathways [69] for gene modules detected by WGCNA. The p-value threshold for enrichment was set to 0.05. The pathways enriched in infectious diseases and cancer were removed.

Monthly DEGs were annotated with Gene Ontology (GO) [70] biological process (BP) and KEGG analysis. The annotations were all achieved with clusterProfiler [71] package, with q-value ≤ 0.05 for GO and p-value ≤ 0.05 for KEGG. Annotation information was retrieved from the Ensembl database using AnnotationHub (https://bioconductor.org/packages/release/bioc/html/AnnotationHub.html) to generate an OrgDb annotation file.

### Pathway-mRNA-lncRNA network construction

We selected genes enriched in key signaling pathways as sources, and co-expressed lncRNA genes as targets to construct the pathway-mRNA-lncRNA network. The Pearson correlations between pathway genes and lncRNA genes were calculated, and those mRNA-lncRNA pairs with correlation higher than 0.8 were selected to construct the network. CytoScape [72] was used to visualize the network.

### Calculation of relative gene expression for monthly DEGs

To observe the effects of melatonin on the expression of representative differentially expressed gene, we plotted relative expression boxplots to visually compare the expression trends of these genes. The horizontal axis of the boxplot represents months, and the vertical axis represents relative expression levels, which were calculated as follows:

$$\varvec{r}\varvec{e}\varvec{l}\varvec{a}\varvec{t}\varvec{i}\varvec{v}\varvec{e} \varvec{e}\varvec{x}\varvec{p}\varvec{r}\varvec{e}\varvec{s}\varvec{s}\varvec{i}\varvec{o}\varvec{n} \varvec{l}\varvec{e}\varvec{v}\varvec{e}\varvec{l} =\mathbf{ln}\left(\frac{\varvec{G}\varvec{e}\varvec{n}\varvec{e} \varvec{C}\varvec{o}\varvec{u}\varvec{n}\varvec{t}\varvec{s} \varvec{i}\varvec{n} \varvec{M}\varvec{e}\varvec{l}\varvec{a}\varvec{t}\varvec{o}\varvec{n}\varvec{i}\varvec{n}}{\varvec{G}\varvec{e}\varvec{n}\varvec{e} \varvec{C}\varvec{o}\varvec{u}\varvec{n}\varvec{t}\varvec{s} \varvec{i}\varvec{n} \varvec{C}\varvec{o}\varvec{n}\varvec{t}\varvec{r}\varvec{o}\varvec{l}}\right)$$

The gene counts were normalized with total counts in different groups. Three relative expression values per month were used for boxplot visualization.

## Supplementary Information

**Additional file 1.** Read counts of all detected genes in 72 samples.

**Additional file 2.** Predicted lncRNAs.

**Additional file 3.** Differentially expressed genes in the control group.

**Additional file 4.** Differentially expressed genes in the melatonin-treated group.

**Additional file 5.** Detailed genes of 3 clusters in the control group (DC1, DC2 and DC3).

**Additional file  6.** The KEGG enrichment results of genes in DC1, DC2 and DC3.

**Additional file 7.** The Pearson correlations between genes and lncRNAs.

**Additional file 8.** Genes with different expression patterns between the control and melatonin-treated groups.

**Additional file 9.** Detailed genes of 3 clusters in the melatonin-treated group (MC1, MC2 and MC3).

**Additional file 10.** The KEGG enrichment results of genes in MC1, MC2 and MC3.

**Additional file 11.** The KEGG analysis results of monthly DEGs.

**Additional file 12.** The GO analysis results of monthly DEGs.

**Additional file 13.** Boxplots of relative expression levels of monthly DEGs.

**Additional file 14.** KEGG Pathway Maps obtained from KEGG (https://www.kegg.jp/kegg/pathway.html).

## Data Availability

All data generated or analyzed during this study are included in this article and Supplementary files, as well as in Genome Sequence Archive (https://ngdc.cncb.ac.cn/gsa/) under accession number CRA005496.

## Abbreviations

SCG: Seasonal changing gene
CAM: Cell adhesion molecule
DEG: Differentially expressed gene
Monthly DEG: Monthly differentially expressed gene
ECM: Extracellular matrix
HF: Hair follicle
SHF: Secondary hair follicle
BMP: Bone morphogenetic protein
SHH: Sonic hedgehog
FGF: Fibroblast growth factor
TGF: Transforming growth factor
KRT: Keratin
KRTAP: Keratin-associated protein
lncRNA: Long noncoding RNA
miRNA: MicroRNA
WGCNA: Weighted gene co-expression network analysis
KEGG: Kyoto Encyclopedia of Genes and Genomes
GO: Gene Ontology
BP: Biological process
PCA: Principal components analysis
LEF: Lymphoid enhancer binding factor
IL: Interleukin
DKK: Dickkopf
