# Supplementary material for: Time-course RNA-seq analysis reveals stage-specific and melatonin-triggered gene expression patterns during the hair follicle growth cycle in Capra hircus
Source: BMC Genomics. 2022 Feb 16;23:140. doi: 10.1186/s12864-022-08331-z (PMC8848980; doi:10.1186/s12864-022-08331-z)
Supplement: Supplementary file 13 — Additional file 13. Boxplots of relative expression levels of monthly DEGs. [file 12864_2022_8331_MOESM13_ESM.docx]

**Additional File 13**
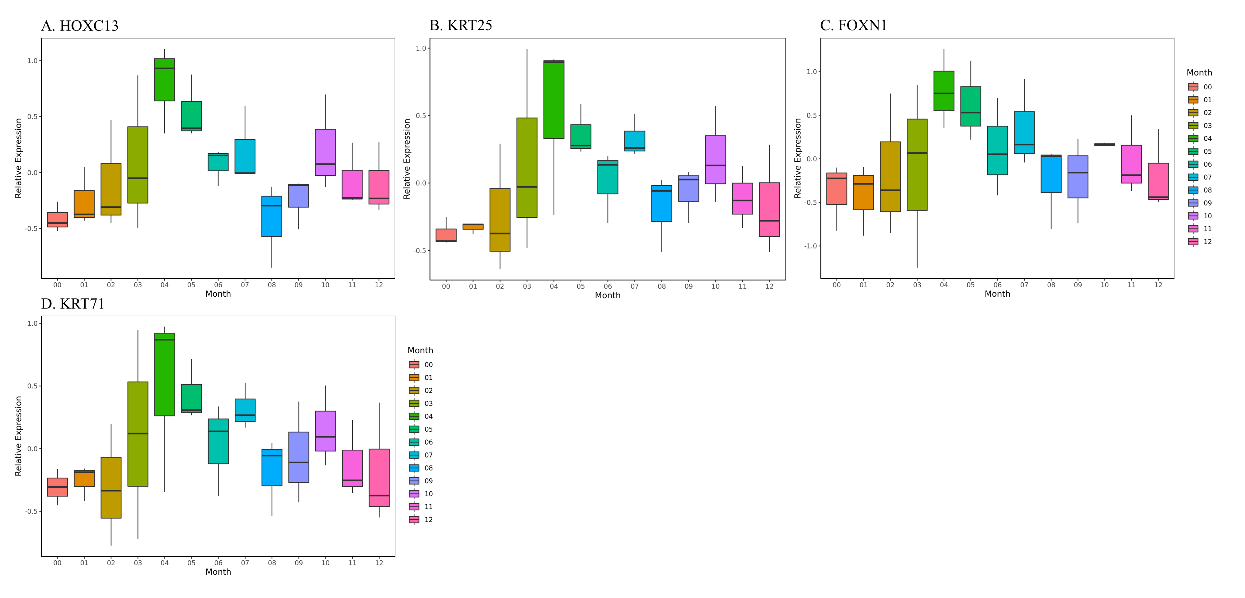


**Figure S1. Boxplots of relative expression levels of HOXC13(A), KRT25(B), FOXN1(c), KRT71(D).** The hair development related genes HOXC13(A), KRT25(B), FOXN1(C), KRT71(D) were highly expressed at the beginning of fast anagen progressing period from April to May, implying that they may be relative to the promotion of fast anagen progressing.


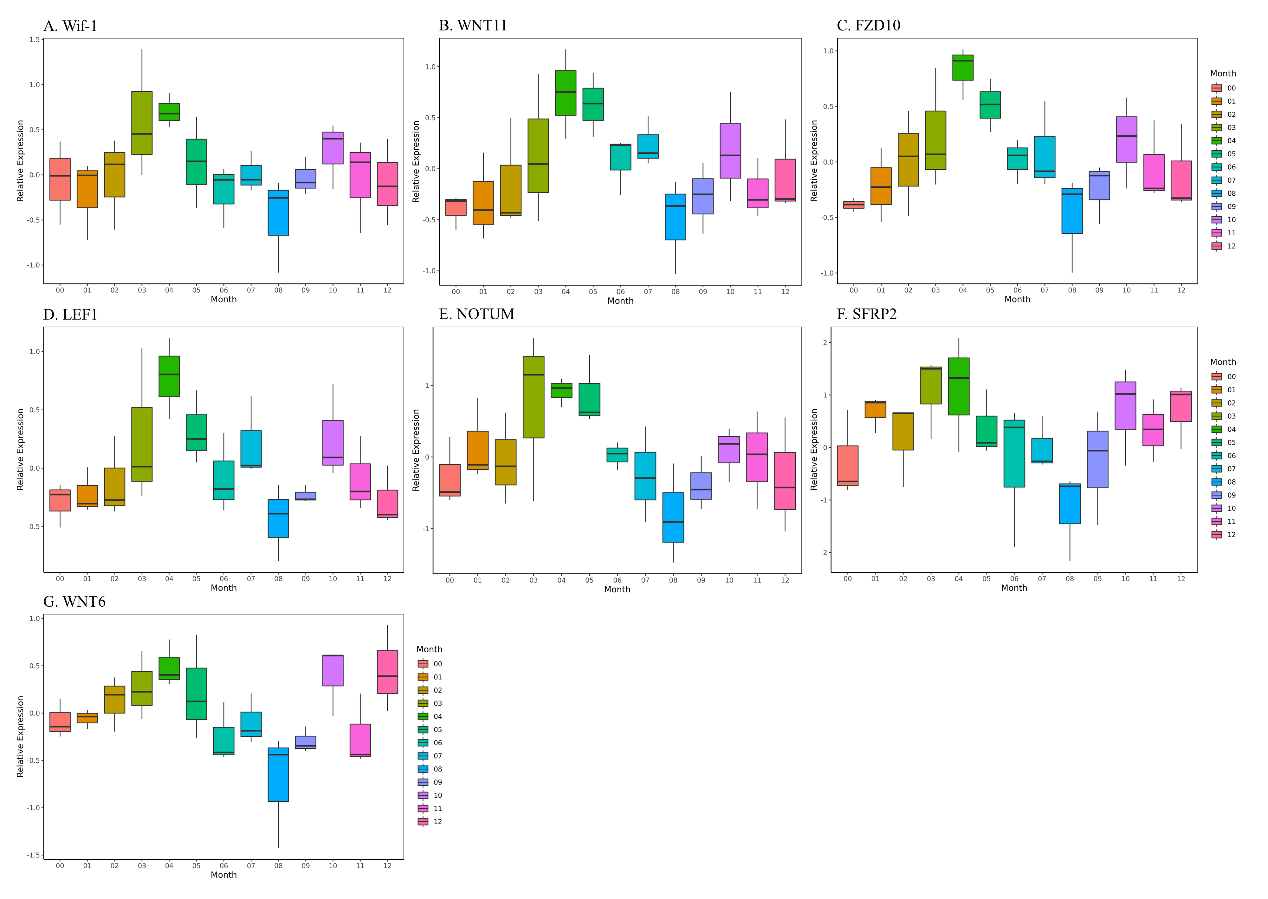


**Figure S2. Boxplots of relative expression levels in WNT signaling pathway (Wif-1(A), WNT11(B), FZD10(C), LEF1(D), NOTUM(E), SFRP2(F), WNT6(G)).** The expression of WNT pathway related genes was generally higher in April and lower in August, implying that WNT related genes may be related to the rapid progression of hair follicles into anagen phase in April and inhibited the growth of hair follicles on the eve of the second cashmere shedding period in August.


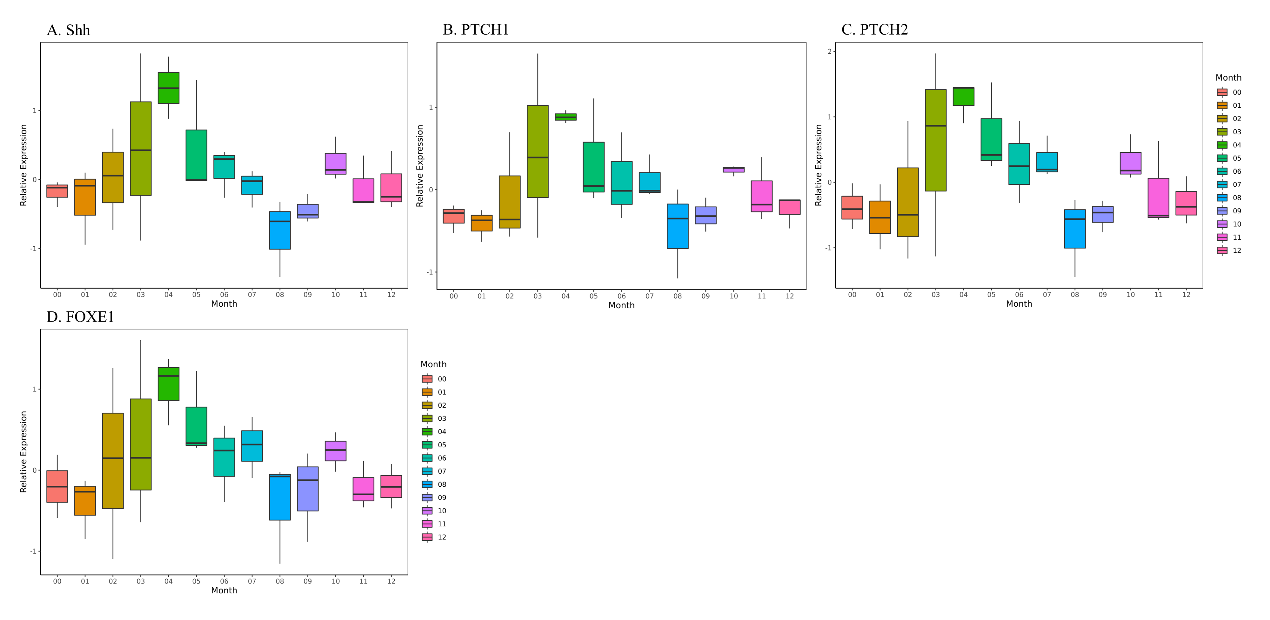


**Figure S3. Boxplots of relative expression levels in Hedgehog signaling pathway (Shh(A), PTCH1(B), PTCH2(C), FOXE1(D)).** The expression patterns of Hedgehog pathway related genes were similar with WNT, with generally higher expression in April and lower expression in August, which similarly implied that Hedgehog related genes may related to promoting anagen transition of hair follicles, whereas they inhibited hair follicle growth on the eve of the second shedding period in August.


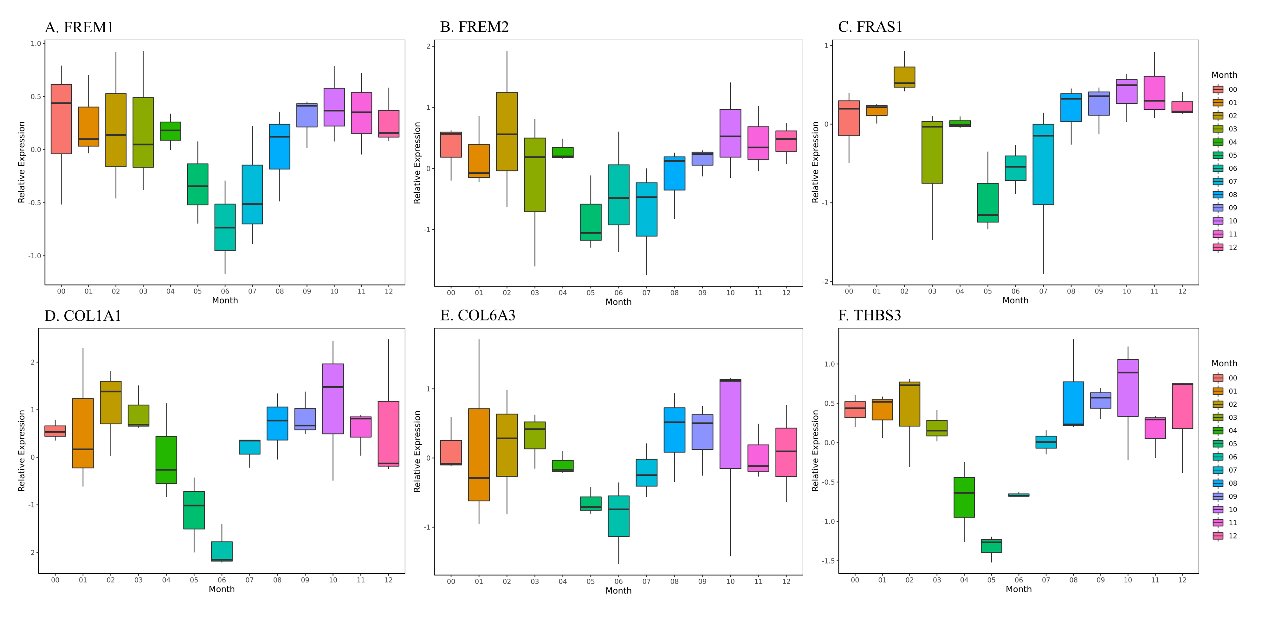


**Figure S4. Boxplots of relative expression levels of ECM related genes (FREM1(A), FREM2(B), FRAS1(C), COL1A1(D), COL6A3(E), THBS3(F)).** The expressions of ECM genes were decreased from May to July. Low expression of these ECM genes may be associated with the second cashmere shedding period.


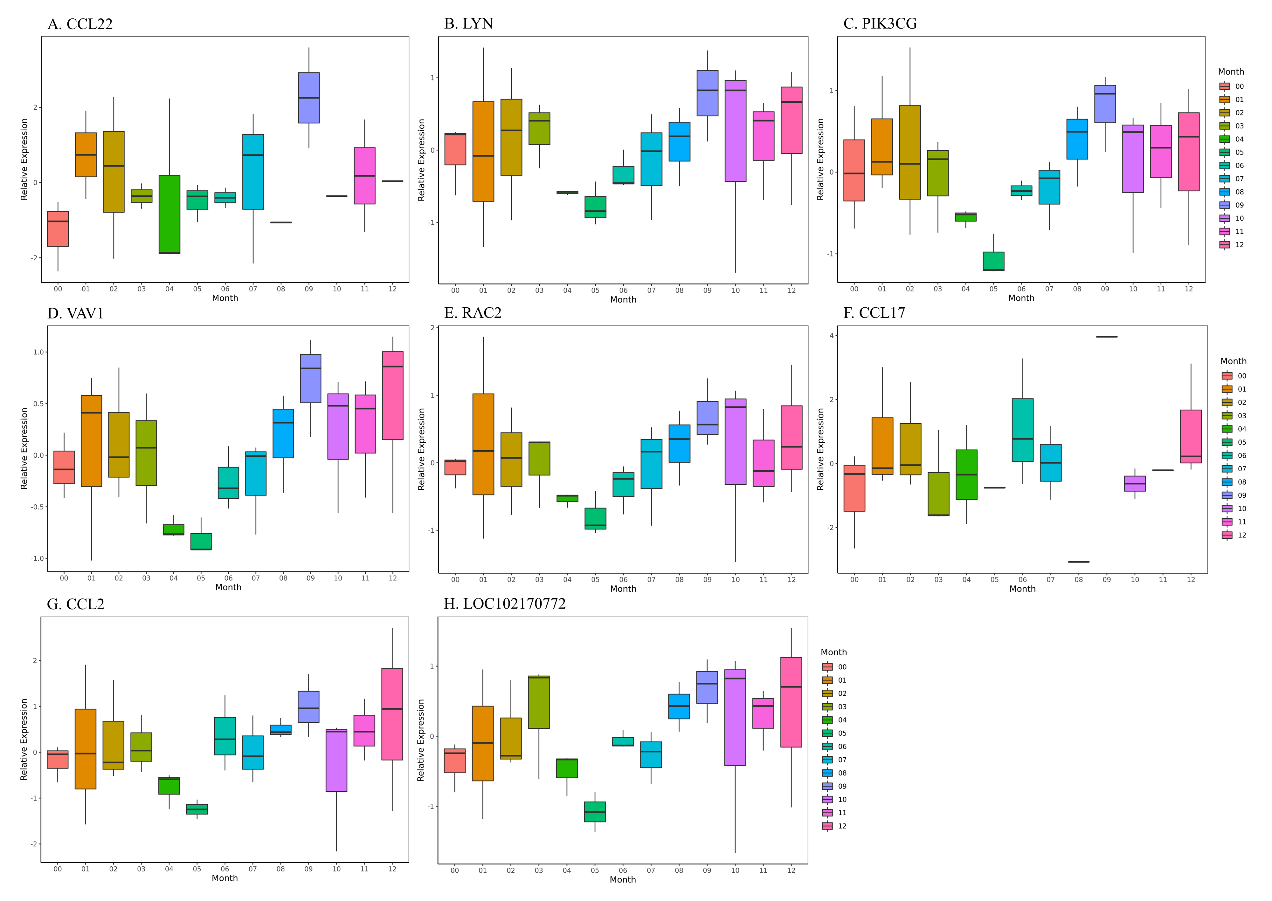


**Figure S5. Boxplots of relative expression levels of Chemokine related genes (CCL22(A), LYN(B), PIK3CG(C), VAV1(D), RAC2(E), CCL17(F), CCL2(G), LOC102170772(H)).** Chemokine genes function in the process of apoptosis. The chemokines were highly expressed from September to October, which may be associated with the second cashmere shedding.


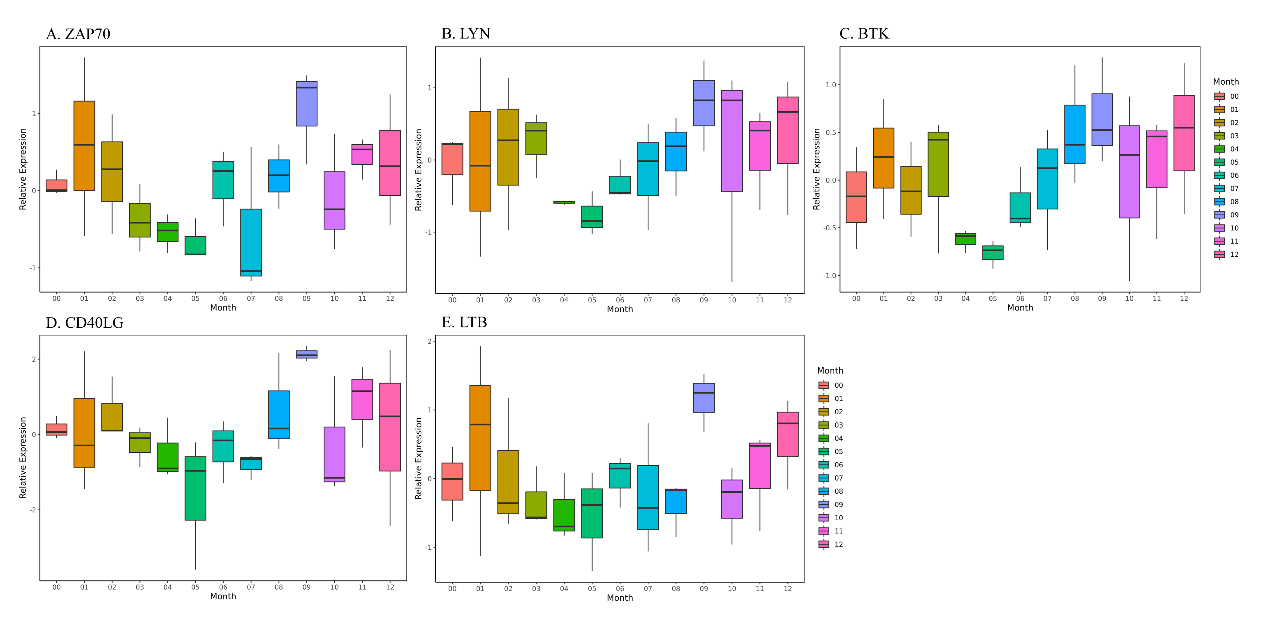


**Figure S6. Boxplots of relative expression levels in NF-κB signaling pathway (ZAP70(A), LYN(B), BTK(C), CD40LG(D), LTB(E)).** These genes were expressed at higher levels in September, which possibly related to the progression of the subsequent cashmere growth. At the same time, LYN in September and October could induce the activation of chemokines, which possibly related to the second cashmere shedding.
