# Supplementary material for: Time-course RNA-seq analysis reveals stage-specific and melatonin-triggered gene expression patterns during the hair follicle growth cycle in Capra hircus
Source: BMC Genomics. 2022 Feb 16;23:140. doi: 10.1186/s12864-022-08331-z (PMC8848980; doi:10.1186/s12864-022-08331-z)
Supplement: Supplementary file 14 — Additional file 14. KEGG Pathway Maps obtained from KEGG (https://www.kegg.jp/kegg/pathway.html). [file 12864_2022_8331_MOESM14_ESM.docx]

**Additional File 14**


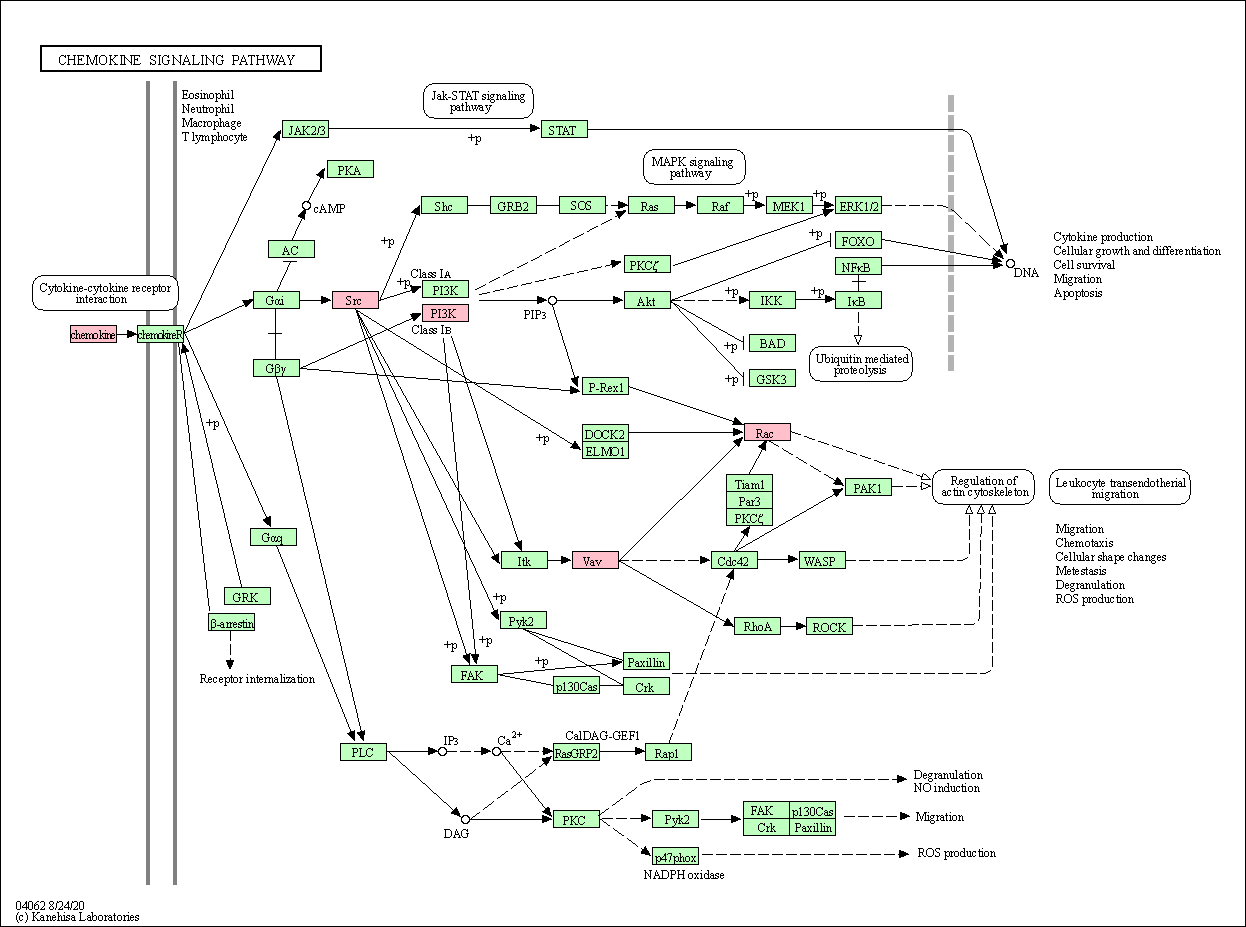


**Figure S1. Chemokine Signaling Pathway virtualized by KEGG Pathway Maps.** High-expressed monthly DEGs are highlighted with pink color, while low-expressed monthly DEGs are highlighted with blue color.


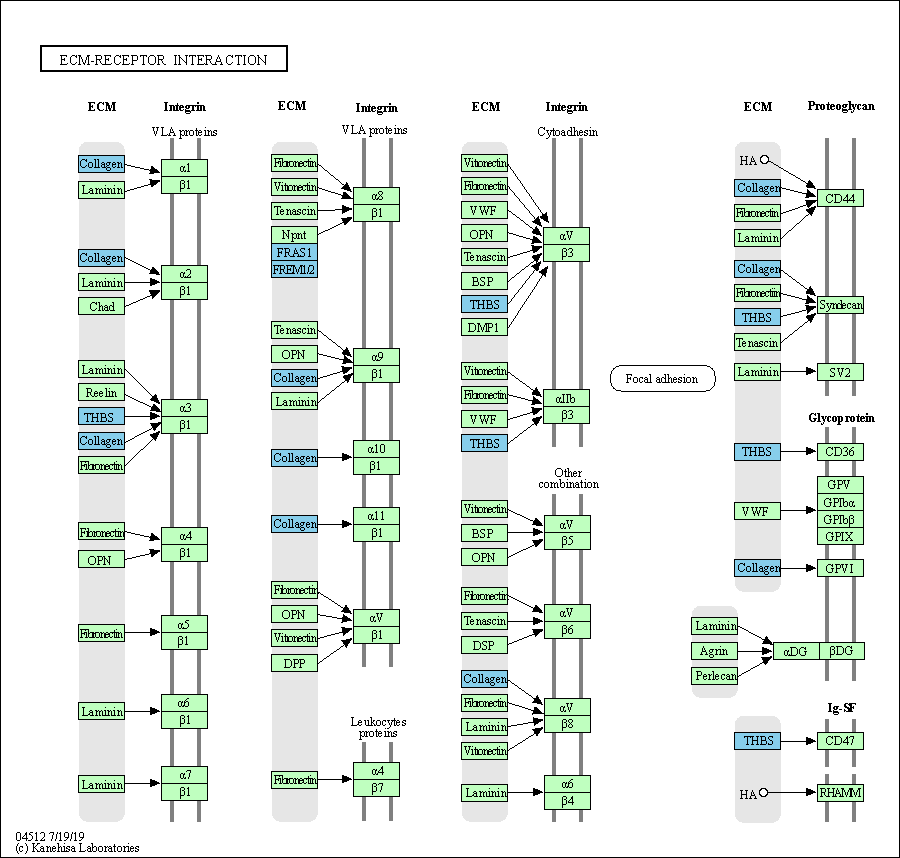


**Figure S2. ECM-Receptor Interaction Signaling Pathway virtualized by KEGG Pathway Maps.** High-expressed monthly DEGs are highlighted with pink color, while low-expressed monthly DEGs are highlighted with blue color.


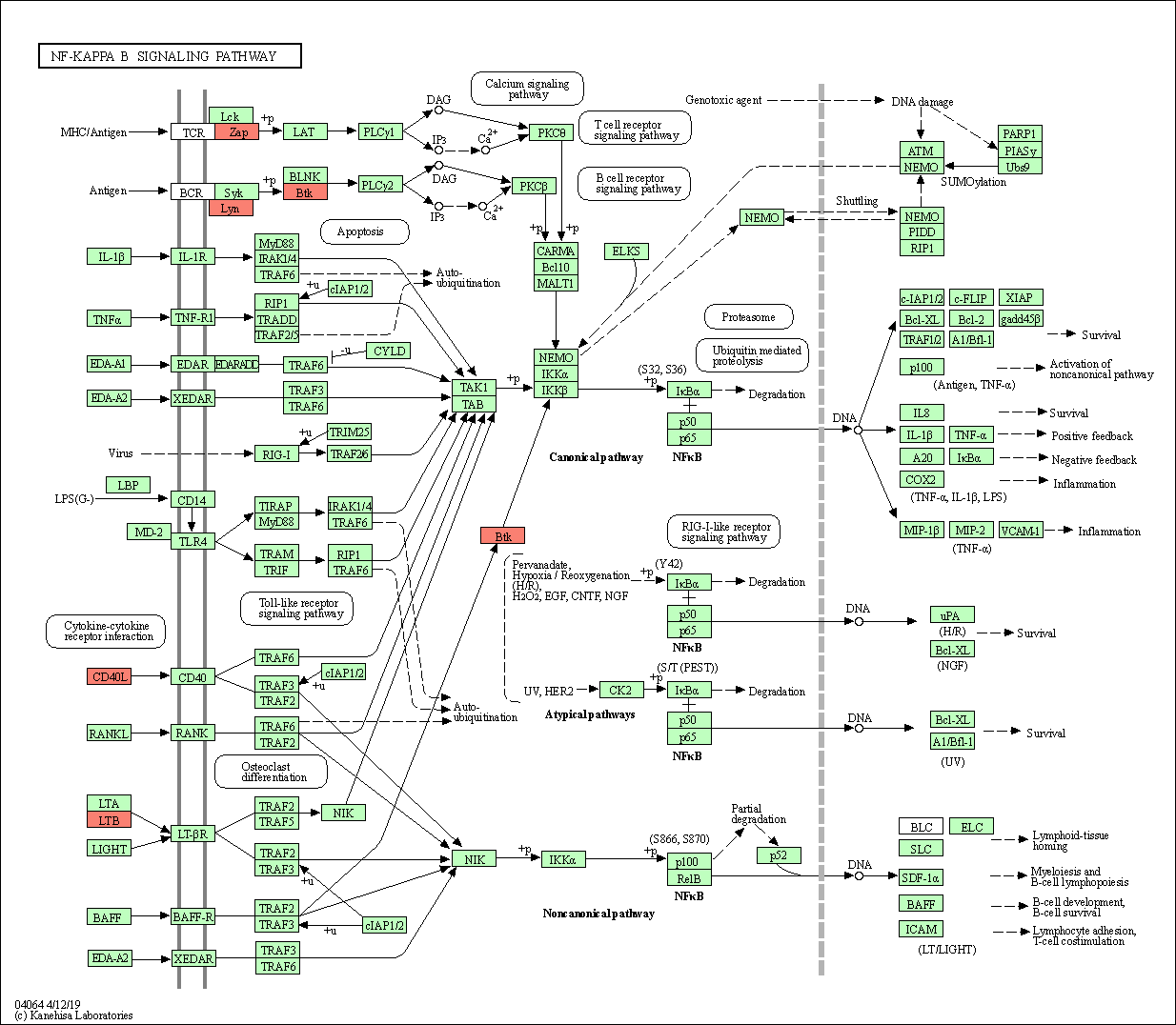


**Figure S3. NF-Kappa B Signaling Pathway virtualized by KEGG Pathway Maps.** High-expressed monthly DEGs are highlighted with pink color, while low-expressed monthly DEGs are highlighted with blue color.


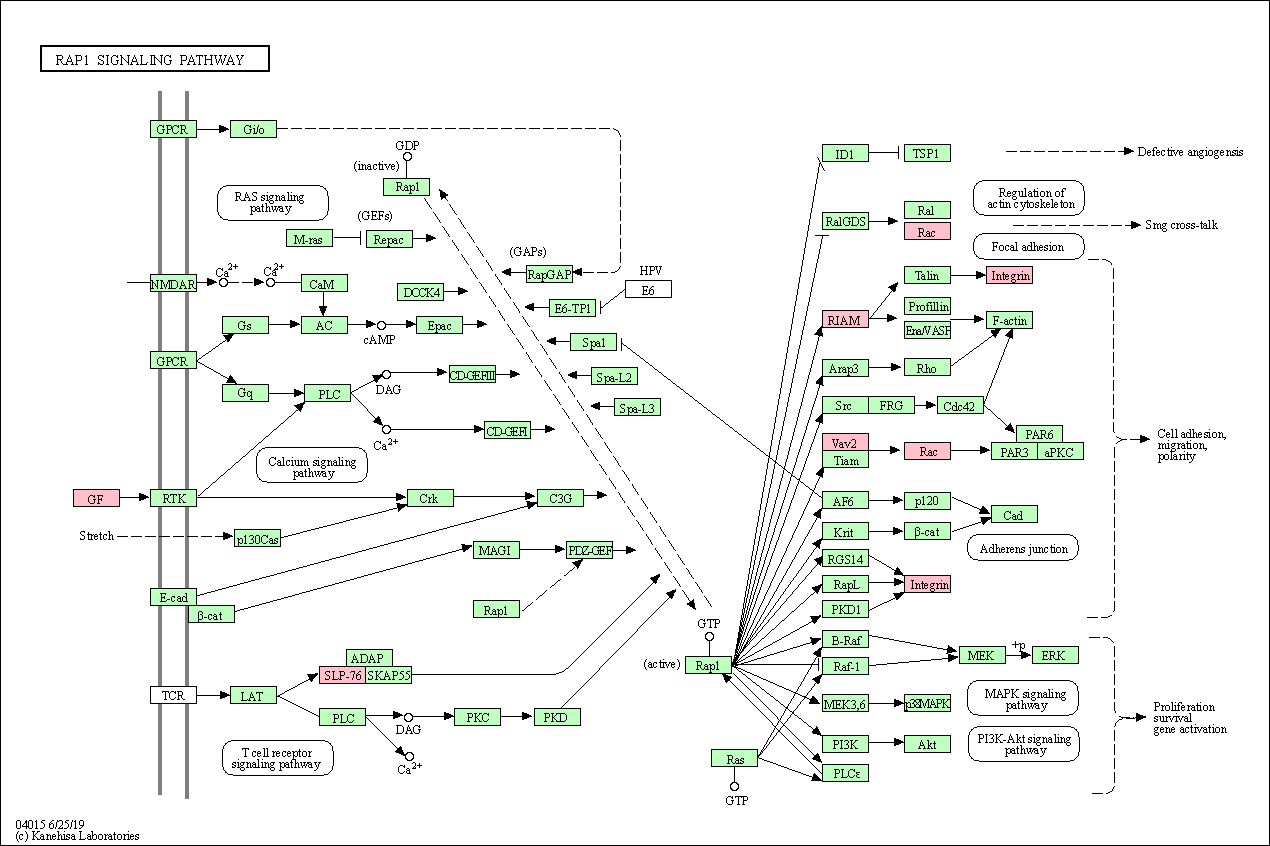


**Figure S4. Rap1 Signaling Pathway virtualized by KEGG Pathway Maps.** High-expressed monthly DEGs are highlighted with pink color, while low-expressed monthly DEGs are highlighted with blue color.


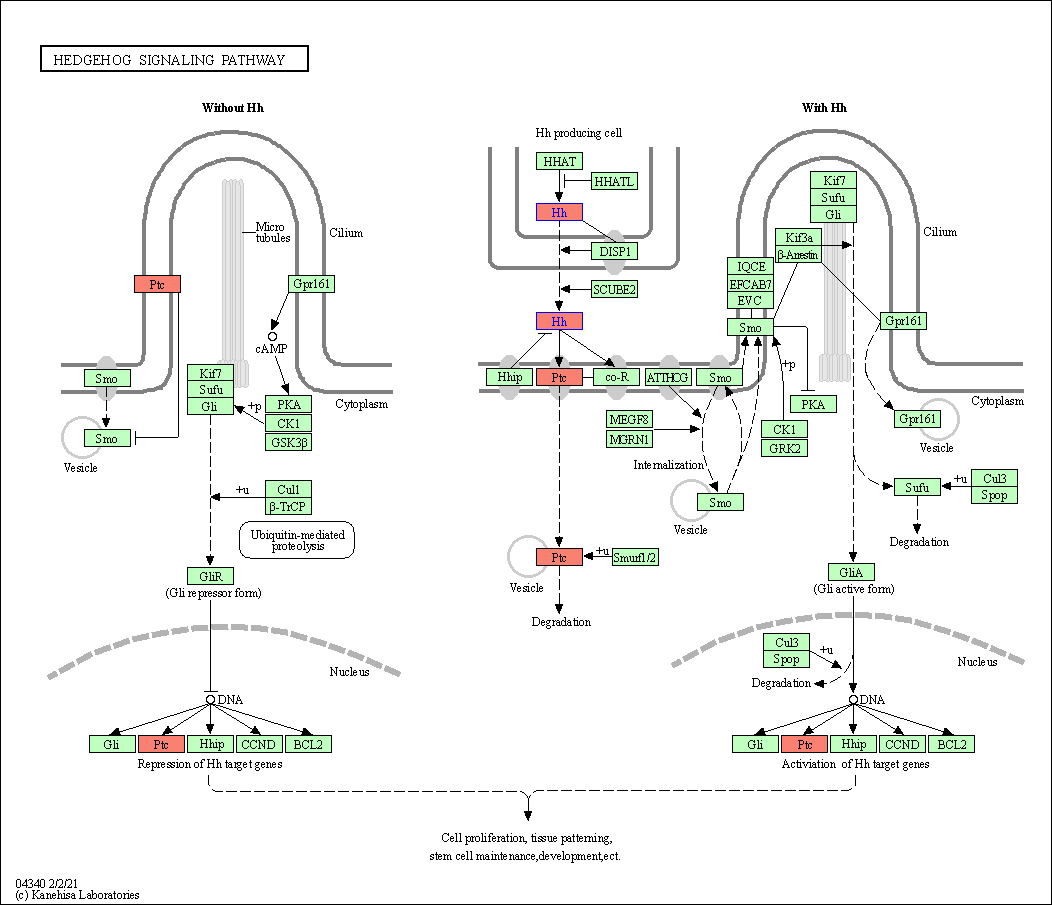


**Figure S5. Hedgehog Signaling Pathway virtualized by KEGG Pathway Maps.** High-expressed monthly DEGs are highlighted with pink color, while low-expressed monthly DEGs are highlighted with blue color.


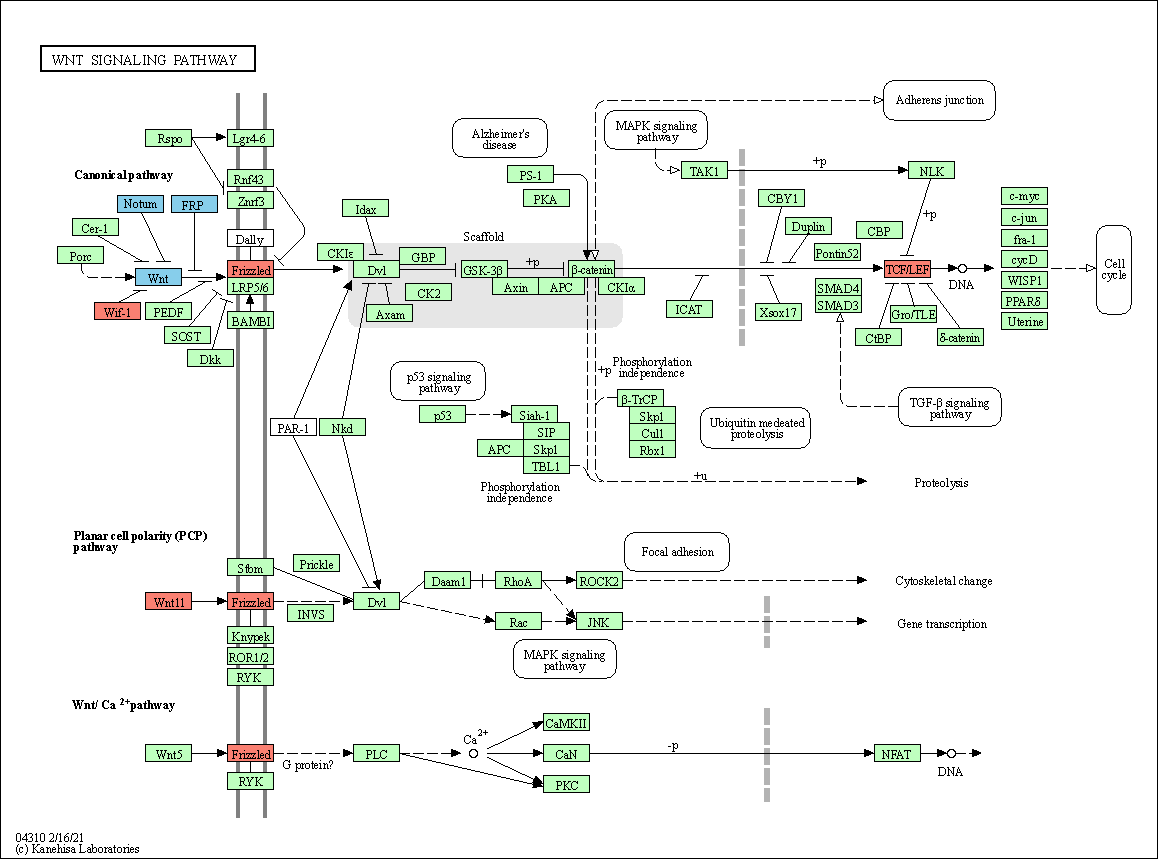


**Figure S6. Wnt Signaling Pathway virtualized by KEGG Pathway Maps.** High-expressed monthly DEGs are highlighted with pink color, while low-expressed monthly DEGs are highlighted with blue color.
